# Renal Water Conservation and Plasma Creatinine in Colorectal Cancer Surgery: A Single-Group Clinical Study

**DOI:** 10.3389/fmed.2022.837414

**Published:** 2022-05-31

**Authors:** Yuhong Li, Rui He, Shuangyan Hu, Robert G. Hahn

**Affiliations:** ^1^Department of Anesthesiology, Shulan (Hangzhou) Hospital, Shulan International Medical College, Zhejiang Shuren University, Hangzhou, China; ^2^Department of Anesthesiology, Shaoxing People's Hospital, Shaoxing, China; ^3^Research Unit, Södertälje Hospital, Södertälje, Karolinska Institutet at Danderyds Hospital (KIDS), Stockholm, Sweden

**Keywords:** creatinine, plasma, urine, Acute Kidney Injury, physiology, dehydration, hydration, gastrointestinal recovery time

## Abstract

Elevation of plasma creatinine after surgery is associated with postoperative morbidity and mortality. We studied whether lengthy surgery might induce renal conservation of water strong enough to exceed the renal capacity to excrete creatinine. Colorectal cancer surgery was performed via laparoscopy in 126 patients. Blood and urine were sampled before surgery, in the postoperative care unit, and 1 day after surgery. The Fluid Retention Index (FRI), which is a composite index of renal water conservation, was calculated. The mean FRI before surgery was 2.4, indicating that patients were well-hydrated. The FRI increased to 2.8 after surgery, and further to 3.5 on the first postoperative day. Plasma creatinine increased in 66 (52%) of the patients while plasma proteins were diluted by 15%. Patients with urinary creatinine > 8.5 mmol/L before surgery were more likely to later show elevation of plasma creatinine (68 vs. 46%, *P* < 0.04). The final FRI was higher in those with perioperative elevation of plasma creatinine (median 3.7 vs. 3.4; *P* < 0.01) and a similar difference was found for the final urinary creatinine concentration (8.5 vs. 7.2 mmol/L; *P* < 0.01). The gastrointestinal recovery time was prolonged when >2 L of Ringer's had been infused during the surgery. We conclude that colorectal surgery initiated a process of renal water conservation that extended into the postoperative period. The water conservation was more intense and the urinary concentration of creatinine was higher in those who had a perioperative rise in plasma creatinine.

## Introduction

Renal water conservation is reflected by high urinary concentrations of metabolic waste products. Urine creatinine, osmolality, urine-specific gravity and visual assessment of urine color have all been suggested as biomarkers useful to detect dehydration and hypovolemia in sports medicine (1–5) and may be summarized in a new robust summary measure, ‘Fluid Retention Index' (FRI) (5, 6). High levels of these biomarkers are often referred to as ‘concentrated urine' and may have implications for the outcome of acute health care (7, 8).

Concentrated urine is also common in the general population (6) and then indicates low habitual intake of water (9). Three small studies suggest that concentrated urine increases the risk of having post-operative elevation of plasma creatinine (10–12), which is associated with complications and increased mortality (13–15).

The expected change in plasma creatinine during surgery is a decrease due to dilution by the intravenous fluid therapy, provided that the muscle metabolism and the kidneys operate as before the surgery. However, transient elevation of plasma creatinine is still common in the perioperative setting and regarded as Acute Kidney Injury (AKI) Grade I if being 50% or more. The precise mechanism for such elevations is unknown.

The present study investigated the relationship between concentration of the urine and post-operative complications after elective laparoscopic colorectal cancer surgery (*primary outcome measure*). For this purpose, FRI was used to quantify the intensity of the renal water conservation during the perioperative period. Special attention was given to a potential relationship between concentrated urine and post-operative elevations of plasma creatinine (*secondary outcome measure*). The hypothesis was that the urine occasionally becomes concentrated enough to elevate plasma creatinine without having to involve permanent impairment of the ability to concentrate the urine.

We also wanted to confirm a previous finding that a large volume of infused crystalloid fluid prolongs the gastrointestinal recovery time (16). The hypothetic mechanism for such prolongation would be that crystalloid fluid easily accumulates in the loose connective tissue of the intestinal wall where it inhibits motion.

## Materials and Methods

### Patients and Ethical Statement

The present non-randomized observational clinical study was based on 126 patients undergoing colorectal cancer surgery by a laparoscopic technique at Shaoxing People's Hospital in South China. The study had been approved by the Ethics Committee of Shaoxing People's Hospital, PR of China (approval 20180115) and registered at the Chinese Clinical Trial Registry (http://www.chictr.org/en; No ChiCTR1800016510, registered June 5, 2018). The first patient was enrolled on August 9, 2018, and the last on November 28, 2019. The inclusion criteria were ASA Class I-II and body mass index of 18-25 kg/m^2^. Exclusion criteria were urogenital disease and severe cardiac lung, hepatic, or renal disease. Written informed consent was obtained from all patients before starting the study. The study adhered to the STROBE checklist. The patient identities were masked when analyzing the data. No part of the data material has been published elsewhere.

### Procedure

Patients arrived at the operating theatre between 7 and 9 a.m., after having fasted overnight. All patients took a laxative at 6 p.m. the day before the surgery. No premedication was given. Anesthesia was induced with midazolam 50 μg/kg, propofol 1.5 mg/kg, cis-atracurium 0.15 mg/kg, and sufentanil 5 μg/kg, followed by endotracheal intubation and mechanical ventilation. The anesthesia was maintained with propofol 6 mg/kg/h and 1.5 MAC of sevoflurane. Additional cis-atracurium 0.05 mg/kg and sufentanil 2 μg/kg was given as required.

An indwelling catheter was placed in the bladder when anesthesia had just been induced and the urine excreted in the morning before the surgery (mean, 198 mL) was sampled. The urine excreted from the onset of anesthesia up to discharge from the post-operative care unit was measured and sampled. The urine samples were taken from mixed content of the urine bag.

Fluid therapy consisted in lactated Ringer's solution (Pharmacia-Baxter, Shanghai, China) and 6% hydroxyethyl starch 130/0.4 (Voluven, Fresenius Kabi Deutschland GmbH, Bad Homburg, Germany). Routine monitoring included pulse oximetry, heart rate, and electrocardiography. The blood loss was estimated from the amount of blood in suction tubes and weighed sponges at the end of the study.

### Measurements

Blood and urine were sampled when the patient had entered the operating room and anesthesia had just been induced, in the post-operative recovery room just before being returned to the surgical ward, and in the first morning on the day after the surgery.

Blood was analyzed for plasma concentrations of creatinine, C-reactive protein, and the blood urea nitrogen (BUN) at the hospital's Clinical Chemistry Laboratory.

Urine was analyzed for specific gravity (refractometry), creatinine, (enzymatic method), osmolality (freezing point depression). The urine color was assessed by visual estimation via a color chart (2).

Fluid Retention Index (FRI) is a composite measure of renal water retention based on four biomarkers of concentrated urine according to the scheme shown in Table 1. Each of the biomarkers is given a score between 1 and 6 where the score represents either a range of values (osmolality and creatinine) or a specific value (urine-specific gravity and urine color). The mean of the four scores yields the FRI value. Hence:


$$\begin{array}{l} \text{FRI}=(\text{specific gravity score}+\text{osmolality score}\\ \;+\text{creatinine score}+\text{color score})/4 \end{array}$$

These four biomarkers are highly inter-correlated and the scores calibrated into each other based on their relationships in a cohort of healthy volunteers aged between 17 and 60 years (5, 6). The purpose is to create a variable that is less sensitive to outliers, as individual biomarkers may occasionally be distorted due to diet and medication. Not outliers were excluded as we trusted that FRI represents the most unbiased index of renal water conservation.

**Table 1:** Scheme for calculating the Fluid Retention Index (FRI), which is the mean of the scores for 4 urinary indexes of concentrated urine.

**Biomarker**	**1**	**2**	**3**	**4**	**5**	**6**
Specific gravity	≤ 1.005	1.010	1.015	1.020	1.025	1.030
Osmolality (mOsmol/kg)	<250	250-450	450–600	600-800	800-1,000	>1,000
Creatinine (mmol/L)	<4	4-7	7-12	12-17	17-25	>25
Color (shade)	1	2	3	4	5	6

*Specific gravity and color were only measured in these steps. Values at a cut-off for osmolality and creatinine are given an in-between score; creatinine of 7.0 yields the score 2.5*.

The mean arterial pressure (MAP) was measured invasively on a multi-function monitor (Datex-Ohmeda, Hoevelaken, the Netherlands) and stored by DoCare Anesthesia Clinical Information System (Medical System, Shuzhou, China). The “patient mean MAP” is the mean value of all measurements performed in one patient.

Dehydration was also assessed by the ratio of BUN to plasma creatinine (17).

The data on the time for recovery of the gastrointestinal function were retrieved from the medical records. Postoperative complications were interpreted according to definitions used in the International Surgical Outcomes Study (ISOS, see http://isos.org.uk).

### Statistics

Data showing a normal distribution are presented as mean (SD) and differences between groups were evaluated by one-way analysis of variance (ANOVA). Differences between > 2 subgroups were further analysed by using the Scheffé *post-hoc* test.

Data showing a skewed distribution are reported as the median (25th−75th percentile limits) and differences studied by Mann-Whitney's *U*-test.

Changes over time were studied by the Wilcoxon's matched-pair test (two measurements) or Friedman's test (3 measurements).

Differences in incidence data were evaluated by contingency table analysis.

Discrimination thresholds were determined by Receiver Operating Curves (ROC).

Correlations were evaluated by simple and multiple regression analysis.

Power analysis was based on whether high FRI score before and/or after the surgery (4 subgroups) was associated with a perioperative decrease/increase of plasma creatinine. An effect size (w) of 0.30, degrees of freedom = 3, power of 80% and *P* < 0.05 yielded that 122 patients was required (G Power 3.1, freeware from Universität Düsseldorf, Germany).

## Results

### Basic Data

The cohort consisted of 40 females and 86 males with a median age of 64 (25th-75th percentiles, 54-69). The most common pre-operative morbidities were hypertension (35%) and diabetes (3%), which were both treated by daily medication.

Key data on the surgeries are shown in Table 2 and additional biochemical data in Table 3. The operations lasted 3.0 (2.5-4) h. The amount of infused Ringer's was 1.8 (1.5-2.2) L, and all patients except three (due to short surgery) were also given 500 mL of hydroxyethyl starch. The largest blood loss was 300 mL, and 15 patients (12%) received erythrocyte transfusion 250-450 mL. After the surgery ended the patients remained in the postoperative care unit for 57 (20) min [mean, (SD)].

**Table 2:** Key data based on whether the urinary Fluid Retention Index (FRI) just before surgery and on the 1st postoperative day was >3.0 or not.

	**Group 1**	**Group 2**	**Group 3**	**Group 4**	***P*-value**
Preoperative FRI > 3.0	No	No	Yes	Yes	
Day after surgery FRI > 3.0	No	Yes	No	Yes	
*N*	28	56	11	31	
**Preoperative**					
Females (*N*%)	7 (25%)	24 (43%)	4 (36%)	5 (16%)	0.06
Age (years)	63 (10)	63 (10)	54 (13)	60 (12)	0.04
Body weight (kg)	61 (7)	63 (9)	61 (10)	63 (10)	0.73
BMI (kg/m^2^)	22.6 (2.1)	23.4 (2.0)	22.4 (2.8)	22.9 (2.6)	0.39
Hypertension (*N*%)	11 (39%)	22 (39%)	3 (27%)	8 (26%)	0.59
Diabetes (*N*)	0	0	1 (9%)	2 (6%)	0.24
P-creatinine (μmol/L)	70 (13)	69 (13)	73 (15)	70 (11)	0.81
U-creatinine (mmol/L)	4.4 (2.5)	4.0 (2.6)	9.4 (1.2)*	10.4 (4.5)*	0.001
FRI (arbitrary unit)	1.8 (0.6)	1.8 (0.6)	3.6 (0.4)*	3.8 (0.6)*	0.001
**End of Surgery**					
MAP (mmHg)^1^	87 (10)	88 (8)	90 (10)	87 (8)	0.67
Operating time (h)	3.8 (1.4)	3.1 (0.7)	3.4 (0.8)	3.4 (0.8)	0.04
Blood loss (mL)	100 (50–150)	100 (50-100)	50 (25-95)	80 (50-100)	0.15
Infused Ringer's (mL)	2,050 (615)	1,737 (511)	2,059 (523)	1,879 (616)	0.07
Urine volume (mL)	585 (245-775)	500 (305-783)	370 (285-618)	300 (158-595)	0.09
Urine flow (mL/min) ^2^	2.1 (1.0-2.6)	1.9 (1-3.1)	1.8 (1.1-2.2)	1.2 (0.7-1.9)	0.047
Urine / Ringer (%)	26 (14-39)	27 (18-47)	24 (16-29)	21 (11-29)	0.08
U-creatinine (mmol/L)	4.9 (3.3)	3.8 (2.6)	5.0 (2.6)	6.3 (3.0)*	0.002
FRI (arbitrary unit)	2.8 (1.0)	2.4 (0.9)	2.9 (1.0)	3.4 (1.0)*	0.003
**Day After Surgery**					
P-creatinine increase (*N*%)	12 (43%)	27 (48%)	3 (27%)	24 (77%)	0.008
U-creatinine (mmol/L)	4.8 (2.3)	8.6 (1.8)*	4.1 (1.1)	9.0 (2.1)*	0.001
FRI (arbitrary unit)	2.3 (0.7)	4.0 (0.6)*	2.3 (0.6)	4.2 (0.5)*	0.001
C-reactive protein (mg/L)	36 (18-52)	34 (27-56)	26 (18-57)	34 (14-43)	0.53
Nausea, vomiting (*N*%)	5 (18%)	9 (16%)	2 (18%)	5 (16%)	0.99
Pain (VAS)	2.9 (0.9)	2.7 (0.6)	2.8 (0.4)	3.0 (0.8)	0.83
Fever (*N*%)	1 (4%)	4 (9%)	1 (9%)	2 (6%)	0.86
Oral food (days)	3.6 (1.3)	3.6 (2.1)	4.0 (1.5)	3.6 (1.9)	0.91
Intestinal recovery time (days)	1.9 (0.8)	1.6 (0.6)	1.6 (0.9)	1.9 (1.3)	0.24
Airway infection (*N*%)	6 (21%)	16 (29%)	3 (27%)	6 (19%)	0.60
Any infectious sign (*N*%)	8 (%)	19 (%)	5 (45%)	12 (39%)	0.82
Reoperation (*N*%)	0	1 (2%)	0	2 (6%)	0.35
Days in hospital	11.6 (3.1)	11.6 (3.4)	10.8 (1.6)	12.0 (3.9)	0.79

*^a^Based on the Patient Mean MAP, Which Was Measured Every 5 min Throughout all Surgeries*.

*^b^Measured From the Start of Surgery up to the Patient Leaves the Postoperative Care Unit*.

Data are either the mean (SD) and evaluated by one-way ANOVA and the ^*^P < 0.05 by the Scheffé post-hoc test, median (interquartile range) and evaluated by the Kruskal-Wallis test, or incidence data compared with contingency table analysis.

**Table 3:** Biochemical measurements on whole blood or plasma in 126 patients undergoing surgery for colorectal cancer.

	**Preoperative**	**1 day after surgery**	**Ratio**	***P-*value**
B-Hb (g/L)	135 (18)	122 (16)	0.90 (0.09)	0.001
Leucocytes (10^9^ / L)	5.7 (1.6)	11.8 (3.6)	2.1 (0.6)	0.001
P-Glucose (mmol/L)	5.3 (0.8)	7.0 (2.1)	1.33 (0.42)	0.001
C-reactive protein (μg/L)	2 (1-5)	34 (21-49)	16 (6-38)	0.001
P-protein (g/L)	69 (5)	58 (5)	0.85 (0.09)	0.001
P-albumin (g/L)	41 (4)	34 (4)	0.83 (0.09)	0.001
IgG (g/L)	12.0 (2.5)	9.9 (2.1)	0.83 (0.10)	0.001
IgA (g/L)	2.5 (1.0)	2.2 (0.9)	0.86 (0.10)	0.001
IgM (g/L)	1.0 (0.7)	0.9 (0.7)	0.90 (0.37)	0.001
Complement C3 (g/L)	1.1 (0.2)	1.0 (0.2)	0.92 (0.44)	0.001
Complement C4 (g/L)	0.32 (0.07)	0.27 (0.07)	0.87 (0.16)	0.001
SGTP (IU/L)	10.6 (11.5)	20.3 (16.0)	1.13 (0.90)	0.52
SGOT (IU/L)	23.1 (8.8)	24.1 (19.5)	1.09 (0.77)	0.59
Bilirubin				
Total (μmol/L)	13.0 (6.0)	13.8 (6.4)	1.15 (0.51)	0.12
Direct (μmol/L)	4.7 (2.2)	5.6 (2.6)	1.28 (0.60)	0.001
Indirect (μmol/L)	8.2 (4.1)	8.3 (4.2)	1.10 (0.52)	0.90

*Measurements are the mean (SD) or the median (25^th^-75 percentile)*.

### Concentrated Urine vs. Urine Flow

The Fluid Retention Index (FRI) before the surgery was 2.1 (1.3). Patients with FRI values on the high side before surgery (> 3.0; *N*=42) were younger, 58 (12) years vs. 64 (10) years (*P* < 0.01). They also had a lower urine flow during surgery, 1.3 (0.9-2.1) mL/min vs. 2.0 (1.2-2.9) mL/min (*P* < 0.01) and excreted a lower fraction of the infused Ringer fluid up to discharge from the post-operative care unit, 21 (11-29)% vs. 26 (16-42)% (*P* < 0.02). Conversely, urine flows <1 mL/min promoted intensified concentration of the urine during the surgery (median FRI increase by 0.5 vs. 0.25 units, *P* < 0.03)

### Concentrated Urine vs. Complications

Complications were explored after dividing the cohort into four groups depending on whether FRI was high (> 3.0) before surgery and/or remained high (Table 2). The patients in Groups 1 and 3 were able to dilute their urine in response to the infused fluid (approximately 2 L of Ringer's) while this ability was weak Groups 2 and 4.

There were no statistically significant differences between these four groups with regard to nausea and vomiting, pain, food intolerance time, fever, infections, and hospital time. However, differences were found with regard to elevation of plasma creatinine (see below).

There were three re-operations of which all were on patients with a posto-perative FRI score of > 3.0.

### Concentrated Urine vs. Plasma Creatinine

The overall plasma creatinine concentration was unchanged (Table 4) but 66 patients showed an increase of which 20 (16%) was > 10%. The plasma proteins were diluted by 15-18% during the same period of time (Table 3).

**Table 4:** Key measurements in plasma and urine that relate to dehydration.

**Measurement**	**Preoperative**	**Postoperative**	**One day after surgery**	***P*-value**
**Plasma**				
Creatinine (umol/L)	69 (13)		70 (14)	0.52
BUN (mgl/dL)	5.0 (1.4)		4.7 (1.4)	0.02
BUN / creatinine ratio 10^−2^	7.1 (5.9-8.8)		6.3 (5.3-7.8)	0.005
**Urine**				
Fluid Retention Index (FRI)	2.4 (1.2)	2.8 (1.0)	3.5 (1.0)	0.001
Color (grade)	2.1 (1.3)	2.6 (1.6)	3.4 (1.5)	0.001
Specific gravity (no unit)	1.011 (1.007-1.018)	1.019 (1.013-1.029)	1.020 (1.015-1.026)	0.001
Osmolality (mosmol/kg)	420 (254-634)	416 (338-522)	659 (630-785)	001
Creatinine (mmol/L)	6.0 (2.5-8.9)	4.1 (2.3-7.4)	8.0 (5.7-9.7)	001

*Measurements are the mean (SD) or the median (25th -75 percentile) depending on the distribution*.

The patients with FRI > 3.0 after surgery more often showed a perioperative increase in plasma creatinine (59 vs. 39%, *P* < 0.01). As shown in Table 2, an even larger fraction of the patients had a rise in plasma creatinine when FRI exceeded 3.0 both before *and* after the surgery (77 vs. 44%; *P* < 0.002). The numerical increase averaged 6% whereas the other patients showed a decrease by 1% (*P* < 0.01).

No pathological values were found; the highest plasma creatinine before surgery was 99 μmol/L and after surgery it was 102 μmol/L.

### Urinary Creatinine Patterns

The FRI score indicated that the water retention gradually intensified during the perioperative period (Table 4, bottom). By contrast, the BUN/plasma creatinine ratio was low and did not increase.

**Figure 1:**
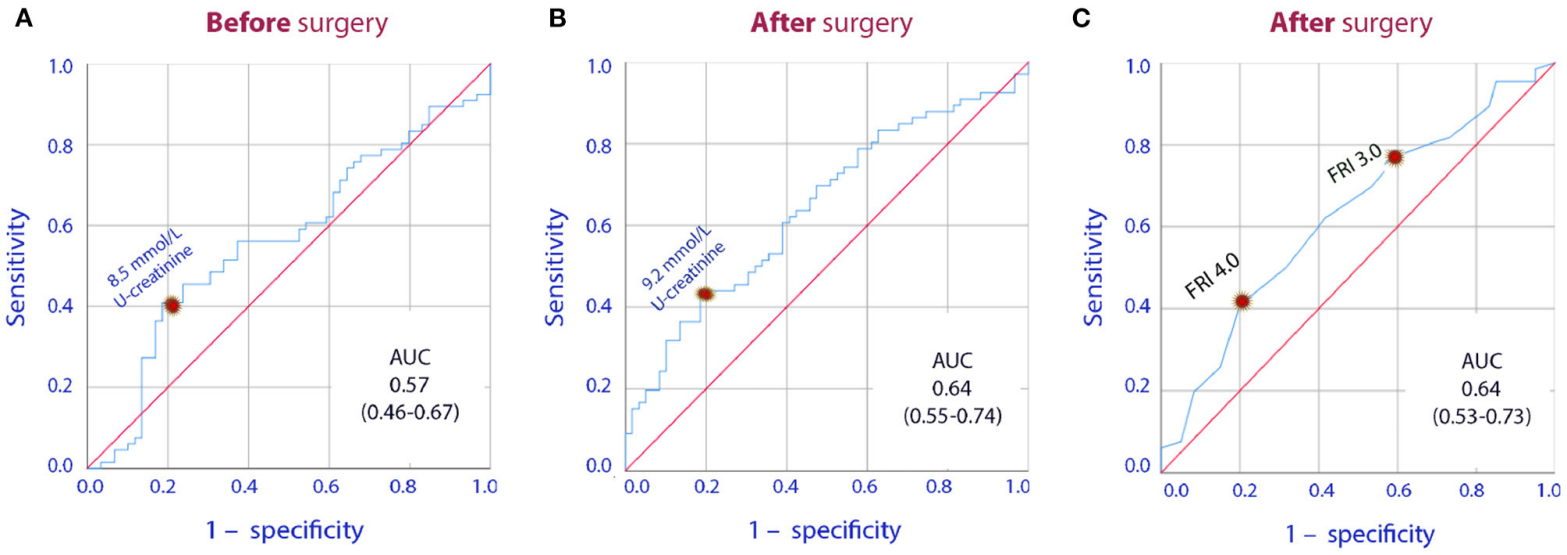
Receiver Operating Curves (ROC) for the U-creatinine concentration measured **(A)** prior to surgery, **(B)** one day after surgery, **(C)** the Fluid Retention Index (FRI) measured one day after surgery when used to indicate whether or not there was a perioperative increase in plasma creatinine. AUC are under the curve followed by the 95% confidence interval is shown.

Despite increasing FRI score, the urinary creatinine concentration decreased temporarily during the surgery (*P* < 0.001; Table 4). Separation of the FRI components depending on the direction of change in plasma creatinine showed that increased plasma creatinine was associated with high urinary creatinine both before the surgery and on the 1^st^ postoperative day, while concentrations were similar at the end of surgery (Table 5, Figures 1A,B). After the surgery increasing plasma creatinine was again statistically associated with higher urinary creatinine [8.5 vs. 7.2 mmol/L; *P* < 0.01], which then adequately paralleled the difference in FRI score between the groups [3.7 vs. 3.4; *P* < 0.01] (Table 5, Figure 1C).

**Table 5:** Urinary measurements relating to dehydration depending on whether patients had a perioperative decrease (–) or increase (+) in plasma creatinine.

**Urine measurement**	**Change in plasma creatinine**	**Preoperative**	**Postoperative**	**One day after surgery**
Color (grade).	–	2.0 (1.3)	2.6 (1.6)	3.1 (1.4)
	+	2.2 (1.3)	2.0 (1.5)	4.0 (1.5)^*^
Specific gravity (no unit)	–	1.009 (1.006-1.017)	1.019 (1.014-1.029)	1.019 (1.015-1.026)
	+	1.013 (1.008-1.018)	1.018 (1.012-1.027)	1.021 (1.016-1.026)
Osmolality (mosmol/kg)	–	357 (249-590)	437 (347-552)	635 (539-788)
	+	469 (278-650)	415 (309-499)	674 (517-775)
Creatinine (mmol/L)	–	5.2 (2.2-8.2)	4.5 (2.3-7.4)	7.2 (5.1-9.1)
	+	7.9 (3.2-9.2)	4.0 (2.4-7.6)	8.5 (6.2-9.9)^*^
Fluid Retention Index (FRI)	–	2.3 (1.2)	2.8 (1.1)	3.4 (1.0)
	+	2.6 (1.1)	2.7 (1.0)	3.7 (1.0)^*^

*Measurements are the mean (SD) or the median (25th-75 th percentile) depending on the distribution*.

* *Different from the other subgroup by P < 0.01*.

Overall, patients with increase in plasma creatinine had 25% higher urinary creatinine than the patients who showed a decrease, the mean of the three measurements being 6.9 (5.1-8.1) vs 5.5 (4.6-7.1) mmol/L, respectively, (P = 0.042).

### Plasma Creatinine vs. Arterial Pressure

The mean patient MAP tended to be slightly lower in patients with an increase in plasma creatinine, 86 (7) vs. 89 (10) mmHg, respectively, (*P* = 0.09) but very low values were rare. The incidence of MAP-values of ≤ 60 mmHg was 1.5% and 0.7% in those with increased and reduced plasma creatinine, respectively. The corresponding incidences of MAP being ≤ 70 mmHg were 11.7 and 9.7%.

### Gastrointestinal Recovery Time

Multiple regression analysis showed that a long gastrointestinal recovery time was associated with a larger volume of infused Ringer's (*r* = 0.35, *P* < 0.001) and advanced age (*P* < 0.03, combined *r* = 0.39) while the operating time was only close to be statistically significant *(P* = 0.07).

Table 6 displays the gastrointestinal recovery times for ranges of infused Ringer volumes (ANOVA *P* < 0.03, post-hoc Scheffé test *P* < 0.05 between lowest and highest fluid range).

**Table 6:** Prolongation of the postoperative recovery time for increasing volumes of infused crystalloid fluid.

**Infused Ringer's**	**Intestinal recovery time (days)**	***N***
<1.5 L	1.5 (0.6)	22
1.5-2.0 L	1.7 (0.8)	53
2.0-2.5 L	1.7 (0.7)	34
>2.5	2.3 (1.7)	17

**Figure 2:**
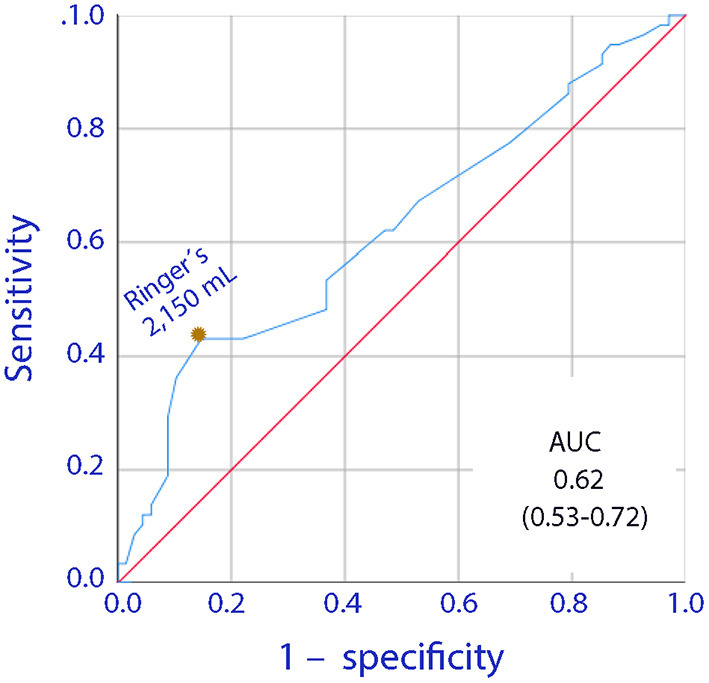
Receiver Operating Curve (ROC) for the relationship between the volume of infused lactated Ringer's during surgery and a postoperative gastrointestinal recovery time of 2 days or more.

Infusion of 2,150 mL of lactated Ringer's was the optimal cut-off point for the fluid-associated increase in the risk of having a recovery time of 2 days or more (Figure 2).

## Discussion

### Key Results

Colorectal surgery initiated a process of renal water conservation that continued into the post-operative period. Table 2 shows that 33% of the cohort had renal water conservation consistent with dehydration before the surgery (FRI > 3.0) while this fraction increased to 69% in the first postoperative morning.

Patients with strong water conservation on the first day after the surgery were more likely to show a rise in plasma creatinine. The reported changes are small but may contribute to our understanding of why plasma creatinine often becomes elevated after surgery. Modest post-operative elevations of were not a sign of permanent poor ability to concentrate the urine, as the patients did so quite effectively very soon after the surgery ended.

The present study did not show any correlation between renal water retention, as evidenced by concentrated urine, and post-operative complications. However, our data suggest a link between concentrated urine and perioperative elevations of plasma creatinine. Concentrated urine before the surgery promoted low urinary flow rates (<1 mL/min), which is known to further concentrate the urine despite adequate fluid administration and MAP (18). The resulting elevated Fluid Retention Index (FRI) score was then statistically associated with perioperative increases of plasma creatinine. The expected change was rather a decrease in line with the dilution of the plasma proteins.

### Urinary Creatinine

Special consideration was given to urinary creatinine, which is one of the four components of the FRI variable that raises plasma creatinine if poorly eliminated by the kidneys. A compelling observation is that patients with a rise in plasma creatinine seemed to strive for a higher urinary creatinine concentration than the others both before and after the surgery but not during the operations. Urinary creatinine even decreased out of proportion of the FRI value, which is a finding also made in a recent study of plasma creatinine in urologic surgery (12).

The urine-specific gravity is regarded to be the most reliable biomarker of renal water conservation (3–5) and increased during the surgery despite the drop in urinary creatinine (Table 5). This discrepancy suggests that something occurring during the anesthesia and surgery transiently reduced the renal concentration capacity for creatinine which, in turn, promoted the postoperative increase of plasma creatinine.

In the normal population, urine creatinine sampled before breakfast seems to reflect the long-term (>1 week) habitual intake of water (9). We hypothesize that kidneys that are “pre-set” to conserve water due to low daily intake of water may require less “stress” or anesthesia-induced water retention to reach the threshold for their ability to further concentrate creatinine. The resulting effect on plasma creatinine is likely to be greater in the presence of an existing limitation of the renal capacity to concentrate creatinine, while acute injury to kidney cells is not a necessary component of the proposed chain of events. A low habitual intake of water combined with sustained renal fluid retention during surgery might then be relevant to plasma creatinine during the perioperative period.

### Fluid Retention Index

We used a relatively new variable to quantify the degree of renal water conservation called Fluid Retention Index (FRI). Our study patients were apparently well-hydrated when surgery started, which may have prevented marked postoperative elevations of plasma creatinine. The Chinese patients scheduled for the surgeries had a starting FRI score of 2.4, which is lower than found in European studies. Healthy Swedish men and women scored 2.8 before and 3.6 after performing recreational physical exercise during 90 min (5). Patients scheduled for abdominal surgery averaged 2.8 (18), as did patients admitted to acute geriatric care (7). 300 hospital workers had 3.7 (6), and hip fracture patients showed 3.7 before surgery (10). The mean FRI value was 3.1 in 187 patients scheduled for before major urological surgery (19).

‘Dehydration' might be defined as FRI ≥ 4.0 (5) but a lower cut-off can be applied to distinguish intense from less intense renal water conservation (20).

A high FRI before surgery is likely to reflect the habitual intake of water (9) and is not strongly age-dependent (5). However, a slight reduction of urine osmolality with age has been demonstrated in large cohorts (21) and is probably due to a gradually impaired ability of the aged kidney to conserve water. Age dependency could be disclosed in the present study, as patients with a high FRI were younger and had lower urine flow rates.

A high ratio of BUN to plasma creatinine indicates impaired blood flow to the kidneys and might be caused by dehydration or congestive heart failure. This ratio correlated poorly to FRI which, however, detects renal conservation of water without direct involvement of the blood flow. The virtually unchanged BUN/creatinine ratio suggests that the kidneys were well perfused before and 1 day after surgery, despite clear evidence of increasing renal fluid retention.

### Hydroxyethyl Starch

Nearly all patients received 500 mL of hydroxyethyl starch during the surgery. This colloid fluid is questioned in Europe due to a risk of kidney injury in septic patients. However, the use of starch during routine surgery does not seem to be associated with kidney injury (22, 23). The present study does not support such concerns either because plasma creatinine was within the normal range. One benefit of starch shown in a previous study from our group is that the colloid does not prolong the gastrointestinal recovery time, while infusing > 2 L of Ringer's did so (16). The present study confirmed our previous finding made about Ringer's (Table 5) while the use of starch was too uniform to be evaluated.

### Limitations

Our report covers perioperative variations in plasma creatinine within the normal range. Only a few of our current findings were convincingly strong, despite being statistically significant. A recent study with greater post-operative elevations of plasma creatinine after urologic surgery showed similar relationships between creatinine in plasma and urine (12) but further studies on patients who fulfil the criteria for postoperative AKI are needed to corroborate the results. We still believe that our reported relationships can be generalized and add to other mechanisms that serve to explain why plasma creatinine often increases in the perioperative setting.

The increase of the intra-abdominal pressure induced by pneumoperitoneum limits the renal perfusion in animals (24) and in man (25). The reduction is considered to affect healthy adults only if there is an underlying impairment of renal function. The renal perfusion in our patients should have been well maintained by the routine administration of hydroxyethyl starch despite small blood losses. Nevertheless, the laparoscopic technique probably contributed to the perioperative increase of the FRI.

The kidney function was of concern. Urine albumin was not measured, but albuminuria is an early sign of renal disease. Our hospital routine is to measure NGAL in patients with kidney disease, but no such measurement was performed because kidney disease was an exclusion criterion. Urine flow might have been decreased by kidney stones in some patients. Late stages of hypertension is associated the impaired kidney function and, therefore, key data are reported separately for normotensive and hypertensive patients in Supplemental File 3. In general, hypertensive patients were somewhat older and had higher BMI than normotensive patients, and their urine flow was higher. However, no patient with elevated plasma creatinine was included in the study.

Benefits of our study include well-controlled surgery, small blood loss, and a relatively conform fluid therapy.

## Conclusions

Laparoscopic colorectal surgery induced renal conservation of water that continued into the post-operative period. Patients who developed a perioperative increase in plasma creatinine tended to show stronger water conservation with higher urinary creatinine concentrations both pre and post-operatively. The high post-operative concentrations contradicts that the reduced inability to concentrate creatinine was long-lasting.

### Institutional Review Board Statement

The study had been approved by the Ethics Committee of Shaoxing People's Hospital, PR of China (approval 20180115) and registered at the Chinese Clinical Trial Registry (http://www.chictr.org/en; No ChiCTR1800016510. The first patient was enrolled on August 9, 2018, and the last on November 28, 2019. registered June 5, 2018) before any patient was enrolled.

## Data Availability Statement

The original contributions presented in the study are included in the article/Supplementary Material, further inquiries can be directed to the corresponding authors.

## Ethics Statement

The studies involving human participants were reviewed and approved by Ethics Committee of Shaoxing People's Hospital, PR of China. The patients/participants provided their written informed consent to participate in this study.

## Author Contributions

Funding acquisition and resources and data curation: YL. Project administration: YL, RH, and SH. Investigation: RH and SU. Conceptualization, methodology, software, validation, formal analysis, writing and visualization: RGH. All authors have read and agreed to the published version of the manuscript.

## Funding

This study was supported by funding from the Zhejiang Provincial Department of Science and Technology Fund (Grant Nos. LY21H150001 and LGF19H030011), Zhejiang Provincial Health Committee Fund (Grant No. 2020KY329) and Hangzhou Medical and Health Science and Technology Project (Grant No. B20210683).

## Conflict of Interest

The authors declare that the research was conducted in the absence of any commercial or financial relationships that could be construed as a potential conflict of interest.

## Publisher's Note

All claims expressed in this article are solely those of the authors and do not necessarily represent those of their affiliated organizations, or those of the publisher, the editors and the reviewers. Any product that may be evaluated in this article, or claim that may be made by its manufacturer, is not guaranteed or endorsed by the publisher.
