# Supplementary material for: Renal Water Conservation and Plasma Creatinine in Colorectal Cancer Surgery: A Single-Group Clinical Study
Source: Front Med (Lausanne). 2022 May 31;9:837414. doi: 10.3389/fmed.2022.837414 (PMC9195291; doi:10.3389/fmed.2022.837414)
Supplement: Supplementary file 3 [file Table_3.DOCX]

**Supplemental File 3.**

Key data based on whether the patients were normotensive or hypertensive.

|  | Normotensive | Hypertension^*^ | P-value |
| --- | --- | --- | --- |
| N | 80 | 46 | .78 |
| PREOPERATIVE |  |  |  |
| Females (N, %) | 26 (33%) | 10 (30%) | .06 |
| Age (years) | 59 (11) | 66 (8) | .0001 |
| Body weight (kg) | 62 (8) | 63 (10) | .45 |
| BMI (kg/m^2^) | 22.7 (2.0) | 23.6 (2.0) | .03 |
| P-creatinine (µmol/L) | 68 (12) | 71 (13) | .17 |
| U-creatinine (mmol/L) | 6.6 (4.4) | 5.3 (3.6) | .09 |
| FRI (arbitrary unit) | 2.6 (1.1) | 2.2 (1.1) | .08 |
|  |  |  |  |
| END OF SURGERY |  |  |  |
| MAP (mmHg)^1^ | 88 (8) | 89 (10) | .53 |
| Operating time (h) | 3.4 (1.1) | 3.3 (1.0) | .38 |
| Blood loss (mL) | 100 (50-108) | 60 (50-100) | .47 |
| Infused Ringer´s (mL) | 1,931 (583) | 1,753 (544) | .09 |
| Urine volume (mL) | 400 (222-629) | 550 (300-770) | .21 |
| Urine flow (mL/min) | 1.7 (0.9-2.5) | 2.1 (1.1-2.9) | .11 |
| Urine / Ringer (%) | 23 (11-36) | 28 (19-41) | .06 |
| U-creatinine (mmol/L) | 5.1 (3.0) | 4.3 (3.0) | .15 |
| FRI (arbitrary unit) | 2.9 (1.1) | 2.6 (0.9) | .19 |
| DAY AFTER SURGERY |  |  |  |
| P-creatinine increase (N, %) | 18 (22%) | 5 (11%) | .14 |
| U-creatinine (mmol/L) | 7.6 (2.7) | 7.4 (2.8) | .78 |
| FRI (arbitrary unit) | 3.5 (0.9) | 3.6 (1.1) | .92 |
| C-reactive protein (mg/L) | 32 (17-49) | 36 (29-56) | .07 |
| Nausea, vomiting (N, %) | 11 (14%) | 10 (22%) | .26 |
| Pain (VAS) | 2.7 (0.8) | 2.9 (0.8) | .14 |
| Fever (N, %) | 3 (4%) | 6 (13%) | .05 |
| Oral food (days) | 3.8 (2.0) | 3.3 (1.7) | .04 |
| Intestinal recovery time (days) | 1.8 (1.0) | 1.7 (0.7) | .50 |
| Airway infection (N, %) | 18 (23%) | 13 (28%) | .50 |
| Days in hospital | 11.3 (2.8) | 12.1 (4.0) | .19 |

^*^ Four patients with diabetes were included, two of them had hypertension.
